# Intraguild predation between *Amblyseius swirskii* and two native Chinese predatory mite species and their development on intraguild prey

**DOI:** 10.1038/srep22992

**Published:** 2016-03-14

**Authors:** Yingwei Guo, Jiale Lv, Xiaohuan Jiang, Boming Wang, Yulin Gao, Endong Wang, Xuenong Xu

**Affiliations:** 1 Lab of Predatory Mites, Institute of Plant Protection, Chinese Academy of Agricultural Sciences, Beijing, China; 2 State Key Laboratory for Biology of Plant Diseases and Insect Pests, Institute of Plant Protection, Chinese Academy of Agricultural Sciences, Beijing, China

## Abstract

*Amblyseius swirskii*, native to the east and southeast Mediterranean region, is a successful biological control agent of whiteflies. In this study, we investigated intraguild predations (IGP) between each stage of *A. swirskii* and each stage of two Phytoseiid species that occur in China, *Amblyseius orientalis* and *Neoseiulus californicus*. When there was no whitefly egg provided as the extraguild prey, IGP between *A. swirskii* and *A. orientalis*, and between *A. swirskii* and *N. californicus*, was observed in 10 and 20 out of 35 combinations, respectively. When IGP was observed, *A. swirskii* was the intraguild predator in 70% and 65% cases of *A. orientalis* and *N. californicus* predation, respectively. These results suggest that *A. swirskii* is a more aggressive intraguild predator compared to either *A. orientalis* or *N. californicus*. When whitefly eggs were provided as the extraguild prey, IGP between *A. swirskii* and *N. californicus* decreased greatly, but no significant decrease of IGP was observed between *A. swirskii* and *A. orientalis*. *Amblyseius swirskii* was able to complete development on both heterospecific predatory mites, and both heterospecific predatory mites completed their development on *A. swirskii*. Possible impacts that *A. swirskii* may have on local predatory mite populations in China are discussed.

*Bemisia tabaci* (Gennadius) (Hemiptera: Aleyrodidae) is one of the most important agricultural pests worldwide^1^,^2^,^3^. In China, it occurs in multiple provinces and generally causes ca. 15% yield loss. Severe outbreaks can lead to a 75% yield loss^4^,^5^,^6^. Efforts are being developed to control whiteflies in environmental friendly ways, such as trap cropping^7^, and biological control. Various natural enemies of whiteflies have been evaluated worldwide, including lady beetles, lacewings, parasitoid wasps, and predatory mites^8^,^9^. Among these candidates, *Amblyseius swirskii* (Athias-Henriot) (Acari: Phytoseiidae) shows extremely high biological control potential^2^,^10^, because it has a high population increase rate, great potential in whitefly control, and is easy to be mass produced^11^. This species is native to the east and southeast Mediterranean region^12^, and is commercially available since 2005^13^. Its successes in Europe and North America initiated our intent to introduce this species to China for whitefly control.

However, introduced natural enemies may have non-target effects on native species that share similar biological niches, including risks of reducing or even eliminating populations of native species^14^. For example, *Harmonia axyridis* Pallas (Coleoptera: Coccinellidae), the multicolored Asian lady beetle, was introduced to Europe, North and South America for aphid and coccid control. This species was a strong competitor and quickly became the top predator, with a negative impact on native ladybeetle species, leading to dramatic decline of some native populations. Now this species is considered an invasive alien pest rather than a biological control agent in these regions^15^,^16^,^17^. Introduced and native species not only compete for food resources and refuges, but may also prey on each other, termed as “intraguild predation” (IGP)^18^,^19^. IGP is an important factor that can be used to estimate the negative impact that introduced species have on local competitors^20^, and occurs prevalently in polyphagous phytoseiid mites^21^,^22^. Therefore, it is necessary to investigate IGP between *A. swirskii* and native Phytoseiid species to evaluate the environmental risks of introducing *A. swirskii* to China.

Kuhlmann *et al.*^23^ proposed three principles in selecting native competitors for risk evaluation: (1) ecological similarities, (2) phylogenetic/taxonomic affinities, and (3) safeguard considerations^23^. In this study, *Amblyseius orientalis* and *Neoseiulus californicus* were selected mainly based on the first two principles. *Amblyseius orientalis* (Ehara) (Acari: Phytoseiidae) is a widely distributed native biological control agent of spider mites and whiteflies^24^. *Neoseiulus californicus* (McGregor) (Acari: Phytoseiidae) is a polyphagous species that is widely distributed worldwide. It co-occurs with *A. swirskii* in the Mediterranean region, and the first Chinese native population was discovered in Dinghushan, Guangdong Province in 2011^25^.

Lab experiments were conducted in 10 mm diameter arenas to investigate IGP between *A. swirskii* and *A. orientalis* on one hand, and between *A. swirskii* and *N. californicus* on the other hand. For each pair of species, we estimated IGP levels between each stage of *A. swirskii* and the native species (eggs, larvae, protonymphs, deutonymphs, female adults, and male adults), either without or with *B. tabaci* eggs provided as the extraguild prey. We also estimated the developmental duration and age specific survival of each predatory mite when it fed on its competitor as the intraguild prey.

## Results

### IGP without or with extraguild prey

*A. swirskii* vs. *A. orientalis.* In the absence of extraguild prey, IGP was observed in 10 combinations, among which *A. swirskii* acted as the intraguild predator in 7 combinations. Table 1 summarizes the IGP levels between the two species. When *A. swirskii* was the predator, adult females preyed on all immature stages of *A. orientalis*, adult males preyed on *A. orientalis* protonymphs, and nymphs preyed on *A. orientalis* eggs. In contrast, when *A. orientalis* was the predator, only adult females preyed on *A. swirskii* eggs and larvae, and deutonymphs preyed on *A. swirskii* eggs.

For the combinations with IGP observed, no significant decrease of IGP level was observed overall (t = 1.087, df = 9, P = 0.305) when extraguild prey was provided. Among all the combinations, significant differences of IGP levels were only detected in 2 combinations (*A. swirskii* adult female vs. *A. orientalis* eggs, and *A. swirskii* adult males vs. *A. orientalis* protonymph) (Table 1).

*A. swirskii* vs. *N. californicus.* In the absence of extraguild prey, IGP was observed in 20 combinations, among which *A. swirskii* acted as the intraguild predator in 13 combinations. Table 2 summarizes the IGP levels between the two species. When *A. swirskii* was the predator, its adults preyed on all immature stages of *N. californicus*, except predations of *N. californicus* eggs by *A. swirskii* males was not observed. Nymphs preyed on all younger stages of *N. californicus*. When *N. californicus* was the predator, adult females preyed on all immature stages of *A. swirskii*. Deutonymphs preyed on *A. swirskii* eggs and larvae, and protonymphs preyed on *A. swirskii* eggs. These data suggest that *A. swirskii* consumes more extensive stages of intraguild prey than *N. californicus*. However,, the IGP level between the two species decreased significantly overall (t = 9.538, df = 19, P < 0.001) when extraguild prey was provided. One exception was for the combination of *A. swirskii* adult female and *N. californicus* eggs (Table 2). These results suggest that extraguild prey may be an important factor that affects the intraguild predation between *A. swirskii* and *N. californicus*.

### Development of *A. swirskii*, *A. orientalis* or *N. californicus* that fed on their intraguild prey

Table 3 summarizes the developmental durations of *A. swirskii*, *A. orientalis* and *N. californicus* when they fed on their heterospecific predatory mites. The original record of predatory mite development can be found as Supplementary Table S1. All *A. swirskii* completed their development when fed on either *A. orientalis* or *N. californicus*. In contrast, only 71.1% and 85.5% eggs of *A. orientalis* and *N. californicus* completed their development, respectively, when they fed on *A. swirskii*. Mean developmental duration of *A. swirskii* was longer than both *A. orientalis* and *N. californicus* when using either competitor as the prey. Both the nymph stages and total developmental duration of *A. swirskii* were shorter when fed on *N. californicus* than on *A. orientalis*, suggesting that *N. californicus* is a more appropriate prey for *A. swirskii*.

## Discussion

In the present study, intraguild predations were observed between *A. swirskii* and *A. orientalis*/*N. californicus*. In each combination, both species could serve as the intraguild predator, and was able to complete its development on the intraguild prey. In most cases where IGP was observed, intraguild predator adults or nymphs preyed on younger stages of the intraguild prey, but adult female of *A. swirskii* also preyed on adult male of *N. californicus*.

*Amblyseius swirskii* was the intraguild predator in 70% and 65% IGP cases, when it co-existed with *A. orientalis* and *N. californicus*, respectively. In addition, *A. swirskii* was capable of preying on nymphs of *A. orientalis* and *N. californicus*. Both *A. swirskii* male and female adults preyed on *A. orientalis* nymphs, while predation on *A. swirskii* nymphs by *A. orientalis* was not observed. Both *A. swirskii* male and female adults preyed on *N. californicus* nymphs, and *A. swirskii* deutonymphs preyed on *N. californicus* protonymphs. In contrast, only *N. californicus* female adults preyed on *A. swirskii* nymphs. When *B. tabaci* eggs were provided, significantly lower levels of IGP were observed between *A. swirskii* and *N. californicus*, but not between *A. swirskii* and *A. orientalis*. This implies that *A. swirskii* might not have negative impact on *N. californicus* in the presence of extraguild prey, but might still have negative impacts on *A. orientalis*.

Many studies investigated intraguild predations between phytoseiid mites^21^,^22^,^26^,^27^,^28^,^29^,^30^. Schausberger^22^ reviewed studies on cannibalism between phytoseiid mites and concluded that the degrees of diet specialization of predatory mites are reflected in their performances in cannibalism^22^. In addition, generalist predators have higher preferences for heterospecific immatures than conspecific immatures, while often not for specialist predators^28^. There is an increased positive correlation between the competitiveness of predatory mites and their prey range. McMurtry and Croft^31^ categorized Phytoseiid mites into 4 categories based on their prey. According to their classification, *A. swirskii* belongs to type III (generalist predators), and *N. californicus* belongs to type II (selective predators of tetranychid mites) although it was also questioned whether this species should be classified as a member of type III^31^,^32^. *Amblyseius orientalis* is a native biological control agent in China. Similar to *N. californicus*, it used to be categorized as type II and was widely used in spider mite control in China^33^,^34^, but recent data indicate that this species is more appropriately categorized as type III^24^,^35^. Our interpretation is that type II and type III include phytoseiid species with prey ranges increasing continuously, and the border between the two categories is actually vague. Both *N. californicus* and *A. orientalis* are critical species ranging between type II and type III, and their prey ranges are narrower than *A. swirskii*, which is consistent with our results suggesting *A. swirskii* is a stronger intraguild predator. McMurtry *et al.*^32^ revised the types of lifestyles of phytoseiid mites. In their study, type II remained almost the same, while type III was subdivided according to their habitat, instead of according to the prey range^32^.These data suggest that it will be valuable to further evaluate the prey range of type II and type III species quantitatively, and analyze the correlation between intraguild competitiveness and prey range.

Higher immature survival was observed for *A. swirskii* than *A. orientalis* and *N. californicus*, when they fed on intraguild prey. The developmental duration of *A. swirskii* was 6.38d and 6.02d when fed on *A. orientalis* and *N. californicus*, respectively, both shorter than its developmental duration when fed on *B. tabaci* (6.96d)^36^. The developmental duration of *N. californicus* fed on *A. swirskii* (4.79d) also is shorter than that when they fed on *Tetranychus urticae* (6.46d)^37^. In contrast, the developmental duration of *A. orientalis* on *A. swirskii* (5.38d) is longer than the same on a mixed prey of *Panonychus ulmi* and *Tetranychus viennensis* (4.20d)^38^.

Some previous studies suggest that the quality of intraguild prey is often lower than the quality of extraguild prey^39^,^40^. However, Buitenhuis *et al.*^41^ indicated that intraguild prey might be an equally good or better food source than the extraguild prey (thrips) for both *A. swirskii* and *Neoseiulus cucumeris* (Oudemans)^41^. Similarly, in this study, the intraguild prey appeared to be more suitable food than extraguild prey for *A. swirskii* and *N. californicus*. We observed that all three predatory mite species are able to reproduce when fed on intraguild prey. Further investigation on fecundity of predatory mites on intraguild prey will allow better estimates of how the introduction of *A. swirskii* will impact population dynamics of local species.

Based on our results, *A. swirskii* is a stronger competitor compared either to *A. orientalis* or *N. californicus*. In addition, *A. swirskii* could have greater negative impact on *A. orientalis* than on *N. californicus*. Therefore it is not possible to deduce whether *A. swirskii* and *A. orientalis* populations are able to coexist without negative consequences, based on the coexistence of *A. swirskii* and *N. californicus* in the Mediterranean region. Possible negative impact of *A. swirskii* on *A. orientalis* populations is herein attributed to 3 main concerns: 1) *A. swirskii* is a stronger competitor than *A. orientalis*, 2) extraguild prey does not lead to significant decrease of IGP levels between the two species, and 3) *A. orientalis* is an equally good or better food source for the development of *A. swirskii* than *B. tabaci*. In contrast, *A. swirskii* appears to be a less appropriate prey for *A. orientalis*.

The current study provided evidences of intraguild predations between *A. swirskii* and *A. orientalis*/*N. californicus* under laboratory conditions, which is an early step in investigating interactions between *A. swirskii* and other predatory mite species in China.

IGP between predatory mites will be more complicated in real agroecosystems. For example, IGP may decrease with increasing habitat complexity^42^, and intraguild prey could avoid or escape areas with the intraguild predator^43^,^44^. Therefore, the impact of these factors, such as habitat complexity and dispersal capability of prey and/or predators on intraguild predations can only be estimated. Furthermore, the impact of intraguild predation on extraguild prey, intraguild predator, and intraguild prey should also be linked with other interactions among these species, which also are influenced by the complexity of real agroecosystems. For example, intraguild predations do not always result in reduced biological control efficiency, while predators that do not prey on each other do not always perform better. Previous studies on biological control of whiteflies provided examples on both sides^45^,^46^. The complexity of the agroecosystem will increase when other organisms sharing the same agroecosystems, including secondary prey, parasitoids, and neutral insects, etc., also are involved^45^,^47^,^48^,^49^. Chailleux *et al.*^48^ stated the necessity to enhance the link between community ecology theory and biological control to develop better pest management strategies^49^. In our case, it is necessary to further evaluate potential interactions between *B. tabaci*, *A. swirskii*, native predatory mite species, and the environment comprehensively, and to find the equilibrium between the values and risks of introducing *A. swirskii* to control *B. tabaci* and other whitefly pests.

## Methods

Laboratory experiments were conducted in small arenas to measure IGP levels between *A. swirskii* and *A. orientalis*, and between *A. swirskii* and *N. californicus*, in the presence or absence of an extraguild prey (eggs of *B. tabaci*), with different combinations of predator stages.

### Predatory mites and whiteflies colonies

*Amblyseius swirskii* used in the present study were obtained from a commercial producer (Koppert Biological Systems, The Netherlands) in 2012, and have been reared on *Carpoglyphus lactis* (Linnaeus) (Acari: Carpoglyphidae). *Amblyseius orientalis* and *N. californicus* were obtained from colonies maintained in the Laboratory of Predatory Mites, Institute of Plant Protection, Chinese Academy of Agricultural Sciences. *Amblyseius orientalis* were reared on *C. lactis* and *N. californicus* were reared on *Tetranychus cinnabarinus* (Boisduval) (Acari: Tetranychidae). The colonies were maintained in a climate chamber at 25 ± 1 °C, 80% ± 5%RH and 16L: 8D photoperiod. *Bemisia tabaci* were obtained from Dry-land Farming Institute of Hebei Academy of Agricultural and Forestry Sciences, and were cultured on tobacco plants in climate chambers (22 ± 1 °C, 60% ± 5%RH and 16L:8D photoperiod).

### Experimental arena

Each experimental arena was built with a transparent acrylic board (30 × 20 × 3 mm^3^) with a 10 mm diameter hole in the center. The top and bottom sides were sealed with a rectangular piece of glass (30 × 20 × 1 mm^3^), and a black plastic sheet, respectively. The three layers were tightly clipped on both ends to avoid predatory mites escaping. When estimating IGP levels with extraguild prey, the bottom side was replaced by a bean leaf disc that contained *B. tabaci* eggs, a piece of filter paper soaked with water to keep the leaf disc moist, and another piece of rectangular glass (30 × 20 × 1 mm^3^). Experimental arenas were maintained in a climate chamber at 25 ± 1 °C, 80% ± 5%RH and 16L:8D photoperiod.

### IGP without or with extraguild prey

IGP between *A. swirskii* and *A. orientalis* or *N. californicus* was investigated without or with extraguild prey. For each pair of predators, IGP levels were estimated at 35 combinations, representing full combinations of all stages (eggs, larvae, protonymphs, deutonymphs, female adults, and male adults) of each species, except the combination of eggs of both species. For each combination, 1 individual of each active stage or 5 eggs of each predator were placed in the experimental arena. To avoid hatching or molting of predators during the experimental duration, eggs <12 h old, larvae <1 h old, protonymphs and deutonymphs <3 h old, and newly emerged adults were used. Twenty replicates were prepared for each combination.

Each combination was examined 12 h later, except for the combinations with the larval stage. Due to the short developmental duration of the larval stage (often <12 h), these combinations were observed 6h later. For each treatment, the number of replicates with 1 active stage killed or consumed, or at least 1 egg consumed were recorded as the number of IGP that occurred.

For all the combinations with IGP observed, IGP levels also were estimated with the extraguild prey provided. For each of these combinations, another 20 replicates were prepared, each provided with 20 *B. tabaci* eggs. Based on our observations, twenty whitefly eggs exceed the maximum consumption rates of both predators. For the predator combinations where no IGP was observed, no replicate in the presence of extraguild prey was conducted.

### Development of *A. swirskii*, *A. orientalis* or *N. californicus* that fed on intraguild prey

For each of the three predatory mite species, eggs laid within 6 hours (considered as 3 hours old on average when the experiment started) were collected and reared individually to estimate development on intraguild prey. Based on the results of the IGP experiment, only adult and nymph stages of the three species performed as intraguild predators (Tables 1, 2). Larvae of the three species do not feed on intraguild prey and are able to develop to protonymph stage without feeding. Therefore, intraguild prey were provided and renewed daily after each predatory mite entered protonymph stages.

When *A. swirskii* and *A. orientalis* coexisted, eggs of both species were intraguild prey in IGP cases (Table 1). In contrast, when *A. swirskii* and *N. californicus* coexisted, both eggs and larvae of intraguild prey often were consumed by elder stages of the intraguild predator (Table 2). Therefore, four sets of predatory mite development were estimated: 1) *A. swirskii* on *A. orientalis* eggs (20 eggs per d), 2) *A. orientalis* on *A. swirskii* eggs (20 eggs per d), 3) *A. swirskii* on *N. californicus* eggs and larvae (10 eggs and 10 larvae per d), and 4) *N. californicus* on *A. swirskii* eggs and larvae (10 eggs and 10 larvae per d). Based on preliminary observations, the amount of intraguild prey provided per d exceeded the consumption rate of the intraguild predators.

For each replicate, the experimental arena was checked once every 12 h, with the stage of the intraguild predator recorded, and its stage specific survival estimated. A minimum of 28 replicates completed their development for each treatment. The mean developmental duration of egg to larva, protonymph, and deutonymph stages were estimated based on the individuals that successfully developed to adults.

### Statistical Analyses

In the intraguild predation experiment, the IGP level of each treatment, either with or without *B. tabaci* eggs provided as the extraguild prey, was estimated as the proportion of replicates with IGP observed. For each combination with IGP, IGP levels without and with extraguild prey were compared using a Chi-squared test. The overall IGP level between the two predators without or with extraguild prey was compared with a paired t-test. ANOVAs were conducted to compare the developmental durations of each immature stage among the predatory mite species. Tukey’s HSD tests were used for multiple comparisons. In all analyses, comparisons with p < 0.05 were considered to have statistical significant differences. Statistical analyses were performed using SPSS 18.0.

## Additional Information

**How to cite this article**: Guo, Y. *et al.* Intraguild predation between *Amblyseius swirskii* and two native Chinese predatory mite species and their development on intraguild prey. *Sci. Rep.* **6**, 22992; doi: 10.1038/srep22992 (2016).

## Supplementary Material

Supplementary Information

## Figures and Tables

**Table 1:** IGP levels between *A. swirskii* and *A. orientalis* in the absence or presence of extraguild prey (whitefly eggs).

Predator		Prey		IGP Level (%)		χ^2^	p
Species	Stage	Species	Stage	Without extraguild prey	With extraguild prey		
*A. orientalis*		*A. swirskii*					
	Deutonymph		Egg	20	25	0.14	0.705
	Adult female		Egg	55	25	3.75	0.053
	Adult female		Larva	10	0	2.11	0.147
*A. swirskii*		*A. orientalis*					
	Protonymph		Egg	10	0	2.11	0.147
	Deutonymph		Egg	50	35	0.92	0.337
	Adult male		Protonymph	25	0	**5.71**	**0.017**
	Adult female		Egg	20	0	**4.44**	**0.035**
	Adult female		Larva	35	55	1.62	0.204
	Adult female		Protonymph	35	45	0.42	0.519
	Adult female		Deutonymph	30	45	0.96	0.327

**Table 2:** Levels of IGP between *Amblyseius swirskii* and *Neoseiulus californicus* in the absence or presence of extraguild prey.

Predator		Prey		IGP Level (%)		χ^2^	p
Species	Stage	Species	Stage	Without extraguild prey	With extraguild prey		
*N. californicus*		*A. swirskii*					
	Protonymph		Egg	65	0	**19.26**	**<0.001**
	Deutonymph		Egg	50	0	**13.33**	**<0.001**
	Deutonymph		Larva	20	0	**4.44**	**0.035**
	Adult Female		Egg	100	0	**40.00**	**<0.001**
	Adult Female		Larva	100	10	**32.73**	**<0.001**
	Adult Female		Protonymph	35	5	**5.63**	**0.018**
	Adult Female		Deutonymph	35	0	**8.49**	**0.004**
*A. swirskii*		*N. californicus*					
	Protonymph		Egg	45	0	**11.61**	**0.001**
	Deutonymph		Egg	80	0	**26.67**	**<0.001**
	Protonymph		Larva	30	0	**7.06**	**0.008**
	Deutonymph		Larva	60	5	**13.79**	**<0.001**
	Deutonymph		Protonymph	45	0	**11.61**	**0.001**
	Adult Male		Larva	75	0	**24.00**	**0.001**
	Adult Male		Protonymph	35	0	**8.49**	**0.004**
	Adult Male		Deutonymph	30	0	**7.06**	**0.008**
	Adult Female		Egg	35	15	2.13	0.144
	Adult Female		Larva	100	40	**17.14**	**<0.001**
	Adult Female		Protonymph	100	20	**26.67**	**<0.001**
	Adult Female		Deutonymph	100	5	**36.19**	**<0.001**
	Adult Female		Adult Male	40	0	**10.00**	**0.002**

**Table 3:** Developmental duration (days) and survival (%) of each intraguild predator.

Predator	Prey	Number of eggs that developed to adult	Developmental duration (d)			
			Stage specific survival %			
			Egg-Larva	Protonymph	Deutonymph	Total immature
*A. swirskii*	*A. orientalis* eggs	28	2.78 ± 0.06bc	2.01 ± 0.10b	1.59 ± 0.05b	6.38 ± 0.09d
			100	100	100	100
*A. orientalis*	*A. swirskii* eggs	30	2.70 ± 0.05b	1.50 ± 0.10	1.18 ± 0.06	5.38 ± 0.11
			77.8	91.4	100	71.1
*A. swirskii*	*N. californicus eggs and larvae*	67	2.89 ± 0.01c	1.67 ± 0.06a	1.47 ± 0.03b	6.02 ± 0.07c
			100	100	100	100
*N. californicus*	*A. swirskii eggs and larvae*	48	2.04 ± 0.03a	1.49 ± 0.03a	1.26 ± 0.03a	4.79 ± 0.06a
			98.2	87.0	100	85.5

^*^Means ± SE in the same column followed by different lowercase letters are significantly different with p < 0.05.
