# Supplementary material for: Intraguild predation between Amblyseius swirskii and two native Chinese predatory mite species and their development on intraguild prey
Source: Sci Rep. 2016 Mar 14;6:22992. doi: 10.1038/srep22992 (PMC4789639; doi:10.1038/srep22992)
Supplement: Supplementary Information [file srep22992-s1.pdf]

Intraguild predation between Amblyseius swirskii and two native Chinese predatory mite species and their development on intraguild prey

Yingwei Guo¶, Jiale Lv¶, Xiaohuan Jiang, Boming Wang, Yulin Gao, Endong Wang*, Xuenong Xu*

Abbrevations: As: *Amblyseius swirskii*
Nc: *Neoseiulus californicus*
Ao: *Amblyseius orientalis*

Predator	Prey	Replicate #	Treatment #	Egg stage (d)	Larval stage (d)	Protonymph stage (d)	Deutonymph stage (d)	Total immature stage (d)	Egg to larval stage (d)
As	Nc	1	1	1.875	0.75	2	1.25	5.875	2.625
As	Nc	2	1	1.875	0.75	1.5	1.5	5.625	2.625
As	Nc	3	1	2.125	0.75	1.25	1.5	5.625	2.875
As	Nc	4	1	2.125	0.75	1.25	1.5	5.625	2.875
As	Nc	5	1	2.125	0.75	1.25	1.5	5.625	2.875
As	Nc	6	1	2.125	0.75	2.75	1.5	7.125	2.875
As	Nc	7	1	2.125	0.75	1.25	1.5	5.625	2.875
As	Nc	8	1	2.125	0.75	1.25	1.5	5.625	2.875
As	Nc	9	1	2.125	0.75	2	1	5.875	2.875
As	Nc	10	1	2.125	0.75	1.25	1.75	5.875	2.875
As	Nc	11	1	2.125	0.75	2.25	1.75	6.875	2.875
As	Nc	12	1	2.125	0.75	1.25	2	6.125	2.875
As	Nc	13	1	2.125	0.75	1.25	1.5	5.625	2.875
As	Nc	14	1	2.125	0.75	2.75	1.25	6.875	2.875
As	Nc	15	1	2.125	0.75	2.75	1.25	6.875	2.875
As	Nc	16	1	2.125	0.75	2.25	1.5	6.625	2.875
As	Nc	17	1	2.125	0.75	1.25	1.5	5.625	2.875
As	Nc	18	1	2.125	0.75	1.75	1.25	5.875	2.875
As	Nc	19	1	2.125	0.75	1.75	1	5.625	2.875
As	Nc	20	1	2.125	0.75	1.75	2	6.625	2.875
As	Nc	21	1	2.125	0.5	1.25	1.25	5.125	2.625
As	Nc	22	1	2.125	0.75	1.25	1.5	5.625	2.875
As	Nc	23	1	2.125	0.75	1.25	1.5	5.625	2.875
As	Nc	24	1	2.125	0.75	1.25	1.5	5.625	2.875
As	Nc	25	1	2.125	0.75	1.25	1.75	5.875	2.875
As	Nc	26	1	2.125	0.75	2.25	1.75	6.875	2.875
As	Nc	27	1	2.125	0.75	1.25	1.5	5.625	2.875
As	Nc	28	1	2.125	0.75	2.75	1.5	7.125	2.875
As	Nc	29	1	2.125	1	1.75	1.75	6.625	3.125
As	Nc	30	1	2.125	0.75	2	1.75	6.625	2.875
As	Nc	31	1	2.125	0.75	1.25	1.5	5.625	2.875
As	Nc	32	1	2.125	0.75	1.75	1.25	5.875	2.875
As	Nc	33	1	2.125	1	1.75	1.25	6.125	3.125
As	Nc	34	1	2.125	0.75	1.75	1.25	5.875	2.875
As	Nc	35	1	2.125	0.75	1.25	1.5	5.625	2.875
As	Nc	36	1	2.125	0.75	1.25	1	5.125	2.875
As	Nc	37	1	2.125	0.75	1.25	1.5	5.625	2.875
As	Nc	38	1	2.125	0.75	1.25	1	5.125	2.875
As	Nc	39	1	2.125	0.75	1.25	1.5	5.625	2.875
As	Nc	40	1	2.125	0.75	2.25	1.5	6.625	2.875

As	Nc	41	1	2.125	1	1.5	1.5	6.125	3.125
As	Nc	42	1	2.125	0.75	1.75	1.25	5.875	2.875
As	Nc	43	1	2.125	0.75	1.75	1.5	6.125	2.875
As	Nc	44	1	2.125	0.75	1.25	1.5	5.625	2.875
As	Nc	45	1	2.125	1	1.75	1.75	6.625	3.125
As	Nc	46	1	2.125	0.75	1.25	1.5	5.625	2.875
As	Nc	47	1	2.125	0.75	1.25	1.5	5.625	2.875
As	Nc	48	1	2.125	0.75	1.75	1.25	5.875	2.875
As	Nc	49	1	2.125	0.75	1.25	1.5	5.625	2.875
As	Nc	50	1	2.125	0.75	2.75	1.25	6.875	2.875
As	Nc	51	1	2.125	0.75	1.25	1.5	5.625	2.875
As	Nc	52	1	2.125	0.75	2.25	2.5	7.625	2.875
As	Nc	53	1	2.125	0.75	1.75	1.5	6.125	2.875
As	Nc	54	1	2.125	0.75	1.25	1.5	5.625	2.875
As	Nc	55	1	2.125	0.75	1.25	1.5	5.625	2.875
As	Nc	56	1	2.125	0.75	1.75	2	6.625	2.875
As	Nc	57	1	2.125	0.75	1.25	1.5	5.625	2.875
As	Nc	58	1	2.125	0.75	1.75	1	5.625	2.875
As	Nc	59	1	2.125	0.75	1.75	1.25	5.875	2.875
As	Nc	60	1	2.125	0.75	1.25	1.5	5.625	2.875
As	Nc	61	1	2.125	0.75	2	1.75	6.625	2.875
As	Nc	62	1	2.125	0.75	1.75	1.25	5.875	2.875
As	Nc	63	1	2.125	0.75	1.25	1.5	5.625	2.875
As	Nc	64	1	2.125	0.75	2.75	2	7.625	2.875
As	Nc	65	1	2.625	0.25	1.25	1.5	5.625	2.875
As	Nc	66	1	2.625	0.5	2	1	6.125	3.125
As	Nc	67	1	2.625	0.5	3	1	7.125	3.125
Nc	As	1	2	0.875	0.75	1.25	1	3.875	1.625
Nc	As	2	2	0.875	0.75	1.25	1	3.875	1.625
Nc	As	3	2	0.875	0.25	1.5	1.25	3.875	1.125
Nc	As	4	2	0.875	0.75	1.25	1.25	4.125	1.625
Nc	As	5	2	1.125	0.5	1.25	1	3.875	1.625
Nc	As	6	2	1.125	0.75	1.25	1.5	4.625	1.875
Nc	As	7	2	1.125	0.5	1.25	1.25	4.125	1.625
Nc	As	8	2	1.125	0.75	1.25	1.5	4.625	1.875
Nc	As	9	2	1.125	0.75	1.75	2	5.625	1.875
Nc	As	10	2	1.125	1	1.5	1.25	4.875	2.125
Nc	As	11	2	1.125	1	1.5	1.25	4.875	2.125
Nc	As	12	2	1.625	0.5	1.75	1.25	5.125	2.125
Nc	As	13	2	1.625	0.5	1.5	1.25	4.875	2.125
Nc	As	14	2	1.625	0.5	1.5	1.25	4.875	2.125
Nc	As	15	2	1.625	0.5	1.5	1.25	4.875	2.125
Nc	As	16	2	1.625	0.5	1.5	1.25	4.875	2.125
Nc	As	17	2	1.625	0.5	1.5	1.25	4.875	2.125
Nc	As	18	2	1.625	0.5	1.5	1	4.625	2.125
Nc	As	19	2	1.625	0.5	1.5	1.25	4.875	2.125
Nc	As	20	2	1.625	0.5	1.5	1.25	4.875	2.125
Nc	As	21	2	1.625	0.5	1	1.5	4.625	2.125
Nc	As	22	2	1.625	0.5	1.5	1	4.625	2.125
Nc	As	23	2	1.625	0.5	1.5	1	4.625	2.125
Nc	As	24	2	1.625	0.5	2.5	1.25	5.875	2.125
Nc	As	25	2	1.625	0.5	1.5	1.25	4.875	2.125
Nc	As	26	2	1.625	0.5	1.75	1.75	5.625	2.125
Nc	As	27	2	1.625	0.5	1.5	1.25	4.875	2.125
Nc	As	28	2	1.625	0.5	1.5	1.25	4.875	2.125

Nc	As	29	2	1.625	0.5	1.75	1.75	5.625	2.125
Nc	As	30	2	1.625	0.5	1.5	1.25	4.875	2.125
Nc	As	31	2	1.625	0.5	1.5	1	4.625	2.125
Nc	As	32	2	1.625	0.5	1.5	1.25	4.875	2.125
Nc	As	33	2	1.625	0.5	1.5	1.5	5.125	2.125
Nc	As	34	2	1.625	0.5	1.5	1.25	4.875	2.125
Nc	As	35	2	1.625	0.5	1.5	1	4.625	2.125
Nc	As	36	2	1.625	0.5	1.75	1.25	5.125	2.125
Nc	As	37	2	1.625	0.5	1.5	1	4.625	2.125
Nc	As	38	2	1.625	0.5	1.75	1	4.875	2.125
Nc	As	39	2	1.625	0.5	1.5	1.5	5.125	2.125
Nc	As	40	2	1.625	0.5	1.5	1.5	5.125	2.125
Nc	As	41	2	1.625	0.5	1.5	1	4.625	2.125
Nc	As	42	2	1.625	0.5	1.5	1.25	4.875	2.125
Nc	As	43	2	1.625	0.5	1.5	1	4.625	2.125
Nc	As	44	2	1.875	0.25	1.5	1.25	4.875	2.125
Nc	As	45	2	1.875	0.25	1	1.5	4.625	2.125
Nc	As	46	2	1.875	0.25	1.5	1.25	4.875	2.125
Nc	As	47	2	1.875	0.25	1.5	1.25	4.875	2.125
Nc	As	48	2	1.875	0.25	1.5	1.25	4.875	2.125
Ao	As	1	4	1.125	0.75	3.25	1	6.125	1.875
Ao	As	2	4	1.625	1	1	1	4.625	2.625
Ao	As	3	4	1.625	1	1.25	1	4.875	2.625
Ao	As	4	4	1.625	0.5	1	1.5	4.625	2.125
Ao	As	5	4	1.625	0.5	1	1	4.125	2.125
Ao	As	6	4	1.875	1	1.75	1.25	5.875	2.875
Ao	As	7	4	1.875	0.75	1.25	1	4.875	2.625
Ao	As	8	4	1.875	0.75	1.25	1	4.875	2.625
Ao	As	9	4	1.875	1	2.75	0.5	6.125	2.875
Ao	As	10	4	1.875	0.75	0.5	1.75	4.875	2.625
Ao	As	11	4	1.875	0.75	2	1.25	5.875	2.625
Ao	As	12	4	1.875	1	2	1.25	6.125	2.875
Ao	As	13	4	1.875	0.75	1	1.25	4.875	2.625
Ao	As	14	4	1.875	0.75	1.25	1	4.875	2.625
Ao	As	15	4	1.875	1	1	1	4.875	2.875
Ao	As	16	4	1.875	0.75	1.25	1	4.875	2.625
Ao	As	17	4	1.875	1	2	1.25	6.125	2.875
Ao	As	18	4	1.875	0.75	1	1	4.625	2.625
Ao	As	19	4	1.875	0.75	1.5	1.5	5.625	2.625
Ao	As	20	4	1.875	1	1.75	1.5	6.125	2.875
Ao	As	21	4	1.875	1	1.75	1.5	6.125	2.875
Ao	As	22	4	1.875	1	1.25	1	5.125	2.875
Ao	As	23	4	1.875	1	1.25	2	6.125	2.875
Ao	As	24	4	1.875	1	2	1	5.875	2.875
Ao	As	25	4	1.875	1	2	1.25	6.125	2.875
Ao	As	26	4	1.875	1	1.75	1.25	5.875	2.875
Ao	As	27	4	1.875	0.75	1.25	1	4.875	2.625
Ao	As	28	4	1.875	1	1.25	1	5.125	2.875
Ao	As	29	4	2.125	1	1	2	6.125	3.125
Ao	As	30	4	2.125	0.75	1.75	0.5	5.125	2.875
As	Ao	1	3	1.125	1	3.75	1.25	7.125	2.125
As	Ao	2	3	1.125	0.75	2	2	5.875	1.875
As	Ao	3	3	1.125	1	2.5	1.5	6.125	2.125
As	Ao	4	3	1.125	1	1.75	2	5.875	2.125
As	Ao	5	3	1.875	0.75	1.25	2	5.875	2.625

As	Ao	6	3	1.875	0.75	1.5	1.75	5.875	2.625
As	Ao	7	3	1.875	0.75	2	1.5	6.125	2.625
As	Ao	8	3	1.875	0.75	2	1.5	6.125	2.625
As	Ao	9	3	1.875	1	1.75	1.5	6.125	2.875
As	Ao	10	3	2.125	0.75	1.75	1.5	6.125	2.875
As	Ao	11	3	2.125	0.75	1.75	1.5	6.125	2.875
As	Ao	12	3	2.125	0.75	1.75	1.5	6.125	2.875
As	Ao	13	3	2.125	0.75	1.75	1.5	6.125	2.875
As	Ao	14	3	2.125	0.75	2.25	1.75	6.875	2.875
As	Ao	15	3	2.125	1	1.75	2.25	7.125	3.125
As	Ao	16	3	2.125	1	2.5	1.5	7.125	3.125
As	Ao	17	3	2.125	0.75	1.75	1.25	5.875	2.875
As	Ao	18	3	2.125	0.75	2	1.75	6.625	2.875
As	Ao	19	3	2.125	0.75	2	1.25	6.125	2.875
As	Ao	20	3	2.125	1	2	1.5	6.625	3.125
As	Ao	21	3	2.125	0.75	2.25	2	7.125	2.875
As	Ao	22	3	2.125	0.75	2	1.75	6.625	2.875
As	Ao	23	3	2.125	0.75	2	1.25	6.125	2.875
As	Ao	24	3	2.125	0.75	1.25	2	6.125	2.875
As	Ao	25	3	2.125	0.75	3	1.25	7.125	2.875
As	Ao	26	3	2.125	1	2.5	1.5	7.125	3.125
As	Ao	27	3	2.125	1	1.75	1.25	6.125	3.125
As	Ao	28	3	2.625	0.5	1.75	1.25	6.125	3.125
